# ^18^F-FDG PET/CT findings in nevoid basal cell carcinoma syndrome: a systematic review and a new case report

**DOI:** 10.1186/s12905-024-03145-5

**Published:** 2024-05-28

**Authors:** Jing Zhang, Yonghong Zhang, Yumeng Jiang, Aodi Xu, Yanli Wang

**Affiliations:** 1 Department of PET/CT, Qingdao Central Hospital, No 127. SiLiu Nan Street, ShanDong Province Qingdao City, 266042 China; 2 Department of Burn and Plastic Surgery, Qingdao Central Hospital, ShanDong Province Qingdao, 266042 China

**Keywords:** ^18^F-FDG PET/CT, Nevoid basal cell carcinoma syndrome, Gorlin-Goltz syndrome, Basal cell nevus syndrome, Breast cancer, Case report

## Abstract

**Background:**

To demonstrate and analyze the ^18^F-FDG positron emission tomography/computed tomography (PET/CT) findings in this rare nevoid basal cell carcinoma syndrome (NBCCS).

**Case presentation:**

A 71-year-old woman with the left invasive breast cancer was treated with hormone therapy for six months and underwent the ^18^F-FDG PET/CT examination for efficacy evaluation. ^18^F-FDG PET/CT revealed the improvement after treatment and other unexpected findings, including multiple nodules on the skin with ^18^F-FDG uptake, bone expansion of cystic lesions in the bilateral ribs, ectopic calcifications and dilated right ureter. She had no known family history. Then, the patient underwent surgical excision of the all skin nodules and the postoperative pathology were multiple basal cell carcinomas. Finally, the comprehensive diagnosis of NBCCS was made. The patient was still in follow-up. Additionally, we have summarized the reported cases (*n* = 3) with ^18^F-FDG PET/CT from the literature.

**Conclusions:**

It is important to recognize this syndrome on ^18^F-FDG PET/CT because of different diagnoses and therapeutic consequences.

## Background

Nevoid basal cell carcinoma syndrome (NBCCS), also known as Gorlin-Goltz syndrome (GGS) and Basal cell nevus syndrome (BCNS), is a rare autosomal dominantly inherited disorder, which is characterized by a wide range of developmental abnormalities and the susceptibility to multiple neoplasms [1–3]. The main clinical presentations include multiple basal cell carcinomas, odontogenic keratocysts, palmoplantar pits, ectopic calcification, skeletal abnormalities and the increased risk of medulloblastoma. Lack of awareness of this syndrome among radiologists and clinicians have led to inappropriate management and treatment for this particular group. A literature reported a case of NBCCS who was diagnosed after developing the fifth type of cancer [4].

Up to now, the majority of published articles reported clinical presentations and conventional imaging manifestations, including X-ray plain film, computed tomography (CT) and magnetic resonance imaging (MRI). Compared to conventional imaging, ^18^F-FDG positron emission tomography/computed tomography (PET/CT) could perform whole-body scan and detect more evidences to support the diagnosis of NBCCS. Additionally, as a non-invasive imaging technique, ^18^F-FDG PET/CT could provide both anatomical and molecular metabolic information. However, the imaging characteristics of NBCCS on ^18^F-FDG PET/CT have rarely been described. We reviewed previous studies and found only three case reports demonstrating ^18^F-FDG PET/CT manifestations of NBCCS [5–7].

We recently encountered a case of NBCCS who was diagnosed at the time of ^18^F-FDG PET/CT examination for efficacy evaluation of the left invasive breast cancer. The purpose of this study was to demonstrate the ^18^F-FDG PET/CT findings of NBCCS and raise awareness of this syndrome.

### Case presentation

**Fig. 1:**
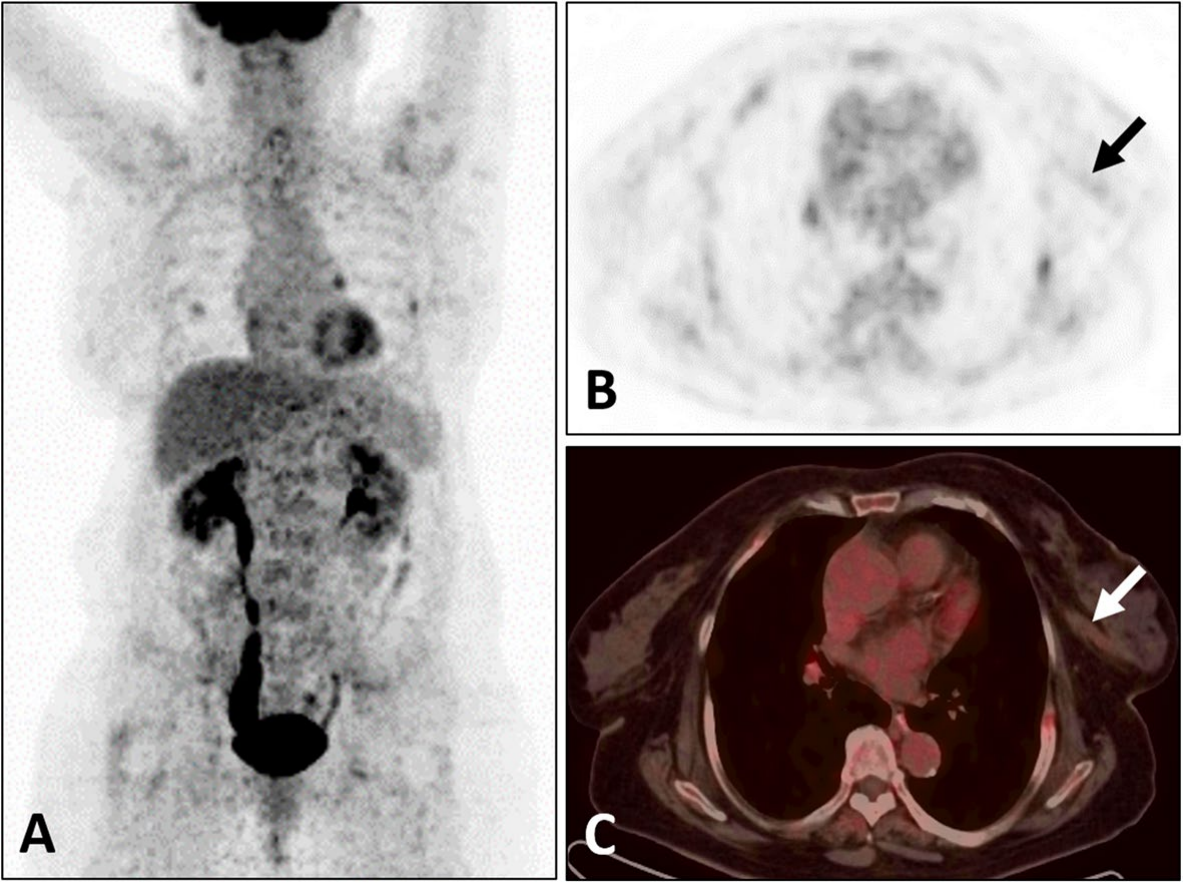
Nevoid basal cell carcinoma syndrome (NBCCS) in a 71-year-old woman with the left invasive breast cancer. The lesion in the left breast (arrow) appeared as strip-like with mildly elevated metabolism of ^18^F-FDG (**A:** MIP; **B:** PET; **C:** PET/CT)

**Fig. 2:**
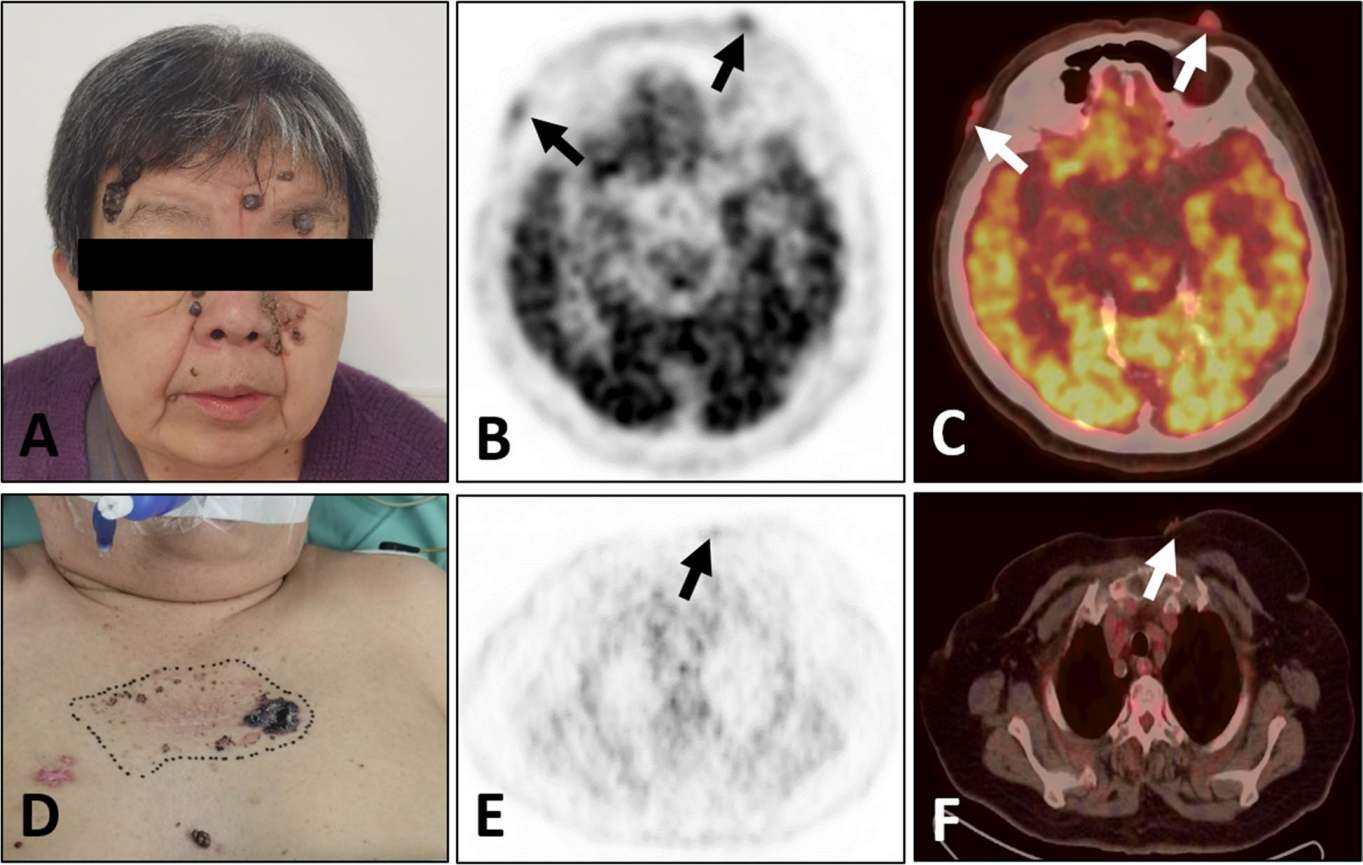
Nevoid basal cell carcinoma syndrome (NBCCS) in a 71-year-old woman with the left invasive breast cancer. There were multiple black and irregular nodules on the head, face and chest wall skin (**A**, **D**). These nodules (arrows) exhibited the different levels of ^18^F-FDG uptake on PET/CT (**B** and **E**: PET; **C** and **F**: PET/CT)

**Fig. 3:**
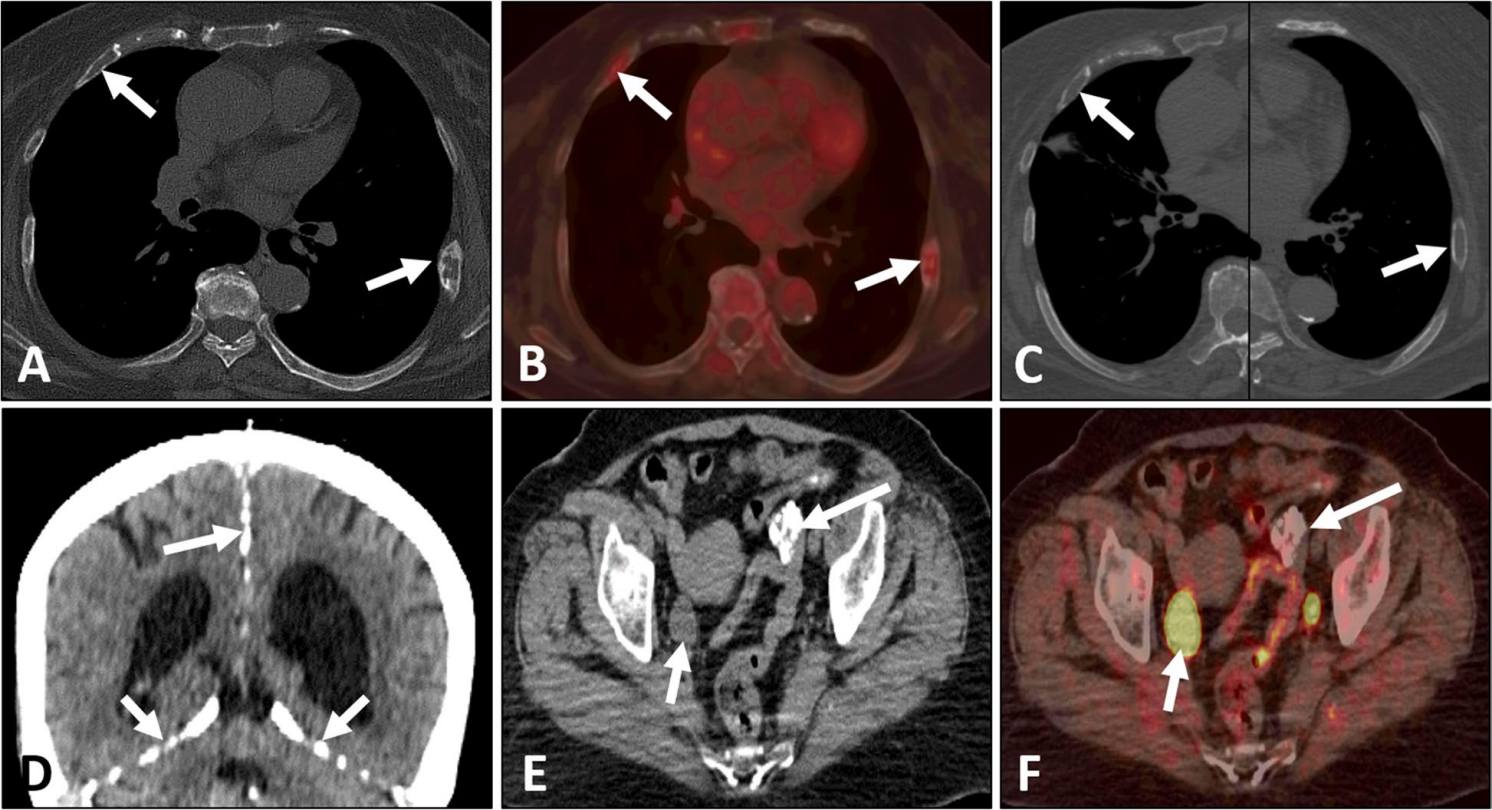
Nevoid basal cell carcinoma syndrome (NBCCS) in a 71-year-old woman with the left invasive breast cancer. The bilateral ribs (arrows) showed bone expansion of cystic lesions and mild thickening of the bone cortex with increased ^18^F-FDG uptake on PET/CT (**A**: PET; **B**: PET/CT). Two years ago, the patient's chest CT images showed the bilateral ribs (arrows) had developed cystic changes (**C**: CT). The falx cerebrum (long arrow) and tentorium cerebellum (short arrows) were accompanied with multiple calcifications (**D**: CT). The left ovary (long arrow) also developed multiple calcifications without ^18^F-FDG uptake (**E**: CT; **F**: PET/CT). The right ureter (short arrow) was dilated (**E**: CT; **F**: PET/CT)

**Fig. 4:**
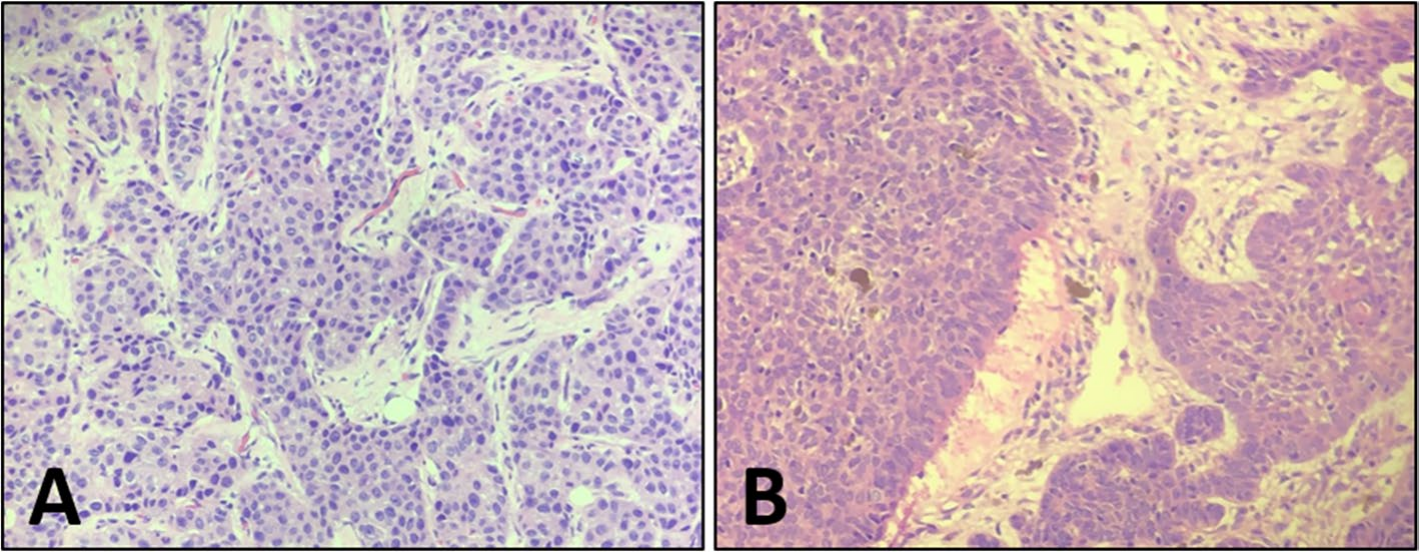
Puncture biopsy of left breast showed invasive carcinoma with histologic grade II. (**A**, hematoxylin–eosin stain × 20). Pathologic findings of the excised skin lesions showed basal cell carcinomas with breakdown and abscess formation, infiltrating the dermis (**B**, hematoxylin–eosin stain × 20)

A 71-year-old women, with a biopsy-proven case of the left invasive breast cancer, received the hormone therapy (exemestane, 25 mg/day) for six months. Patient self-reported a history of resection for multiple basal cell carcinomas of the skin and received radiation therapy for twenty years. Enlarged skin nodules with itching symptom in the last five years. She denied fever, weight loss and other obvious discomfort symptoms. Laboratory result was notable for increased white cell counts 651.93/uL (reference range, 0.00–25.00/uL) in the routine urine testing. The levels of all tumor markers, including carcinogenic embryonic antigen (CEA), cancer antigen153 (CA153) and cancer antigen 125 (CA125), were within the normal range. ^18^F-FDG PET/CT revealed that the breast lesion was significantly reduced in volume and slightly increased in ^18^F-FDG metabolism [Maximum standardized uptake value (SUVmax), 1.6] (Fig. 1), which indicated the improvement after treatment. There were multiple black and irregular nodules on patient’s head (Fig. 2A), face (Fig. 2A) and anterior chest skin (Fig. 2D). These nodules exhibited different levels of ^18^F-FDG uptake on PET/CT (Fig. 2B-C and Fig. 2E-F). The highest level of ^18^F-FDG uptake was found on patient’s face skin (SUVmax, 6.2). Meanwhile, the bilateral ribs showed bone expansion of cystic lesions and mild thickening of the bone cortex with increased ^18^F-FDG metabolism (SUVmax, 3.9) on PET/CT (Fig. 3A,B). When we reviewed her chest CT images from two years ago, the bilateral ribs have developed cystic changes (Fig. 3C). Currently, the volume slightly increased compared by before. Other interesting findings included ectopic calcifications in the falx cerebrum (Fig. 3D), tentorium cerebellum (Fig. 3D) and left ovary (Fig. 3E). There was no significant abnormal ^18^F-FDG metabolism in the region of left ovary (Fig. 3F). The right ureter was dilated (Fig. 3E,F), which was related with history of pyelonephritis. The patient underwent surgical excision of the all skin nodules. The postoperative pathology were multiple basal cell carcinomas. Pathologic findings of breast cancer and basal cell carcinomas were shown in the Fig. 4A and B, respectively. Finally, comprehensive diagnosis of NBCCS was made. The patient was still in follow-up.

Then, we summarized the prior reported cases of NBCCS with ^18^F-FDG PET/CT presentations. In our review, there were 4 cases in total including our case. Pachowicz et al. [5] reported a 51-year-old patient had multifocal skin basal cell carcinomas. ^18^F-FDG PET/CT revealed complex rib anomalies, scoliosis with vertebral malformations and cysts of jaw. But no ^18^F-FDG avid areas were observed on PET/CT. Yin et al. [6] reported an 18-year-old woman had a surgical history of removing multiple odontogenic cysts. ^18^F-FDG PET/CT showed a cyst in the right maxilla with ^18^F-FDG uptake. MRI of the pelvis demonstrated a large solid tumor of the right ovary and postoperative pathology confirmed the benign ovarian fibroma. Reaz et al. [7] reported a 20-year-old woman underwent a surgery of odontogenic keratocysts. There was a hyperactive mass in the left ventricle and no other hypermetabolic area on whole-body ^18^F-FDG PET/CT. Our case reported multiple basal cell carcinomas with ^18^F-FDG uptake, bone expansion of cystic lesions in the bilateral ribs and ectopic calcifications (the falx cerebrum, tentorium cerebellum and left ovary). And these findings were found during the efficacy evaluation of breast cancer. The ^18^F-FDG PET/CT findings of all cases were detailed in Table 1.

**Table 1:** The ^18^F-FDG PET/CT findings in Nevoid basal cell carcinoma syndrome (NBCCS)

	Pachowicz et al. [5]	Yin et al. [6]	Reaz et al. [7]	Present case
Multiple basal cell carcinomas	former	-	-	+
Odontogenic keratocyst	+	+	former	-
Skeletal anomalies	+	-	-	+
Ectopic calcification	-	-	-	+
Cardiac fibroma	-	-	+	-
Ovarian fibroma	-	+	-	-
Breast cancer	-	-	-	+

+ Positive

-Negative

## Discussion and conclusions

NBCCS is a rare autosomal dominantly inherited disorder and presents as multisystem abnormalities. This disease has the estimated prevalence of 1/57000 to 1/256000 and both sexes are equally affected [8]. The patched-1 (PTCH1) gene, an onco-suppressor gene that maps at 9q22.3 region, is the major causative gene of NBCCS, which involves in the hedgehog signaling pathway [9, 10]. The mutation is transmitted in an autosomal dominant inheritance from parents to their children. However, 35% to 50% of NBCCS are spontaneous mutations and without family history [10].

The diagnostic criteria for NBCCS were first proposed by Evans et al. in 1993 [11]. After the reviewed and revised by Kimonis et al. in 1997 [12], Kimonis et al. [13] in 2004 and Bree et al. in 2011 [14]. A positive diagnosis was established by the presence of one major criterion and genetic confirmation or by two major criteria or by one major and two minor criteria. The major diagnostic criteria would include multiple basal cell carcinomas, odontogenic keratocyst of the jaw, palmar pitting, calcification of the falx cerebri, medulloblastoma and first-degree relatives with NBCCS. And the minor diagnostic criteria would include rib abnormalities, other skeletal malformations and radiologic changes, macrocephaly, lip palate, cardiac or ovarian fibroma, lymphomesenteric cysts and ocular abnormalities. Although the patient in our report denied family history of NBCCS and did not get PICH1 genetic testing, she had multiple basal cell carcinomas, ectopic calcifications and bilateral rib anomalies, which met the diagnostic criteria for NBCCS.

In previously published articles, the authors mostly reported the clinical presentations and conventional imaging manifestations [4, 15–20]. Figueira et al. [21] emphasized that it is essential for dental surgeons and dermatologists to know the signs and symptoms of NBCCS, which is important for patients to get early diagnosis and more rational approach to the treatment. Compared to conventional imaging, PET/CT, as a whole-body examination, had the advantage to find more multisystem abnormalities to support the diagnosis of NBCCS. However, there were only three case reports based on PET/CT scans. Additionally, we were the first to report that breast cancer patient with NBCCS on PET/CT.

Basal cell carcinoma is a relatively inert primary cutaneous neoplasm, which usually invades the local skin and adjacent structures but rarely metastasizes far away. It has also been associated with certain syndromes, the NBCCS being the most common [22]. Ayala et al. [23] held the view that ^18^F-FDG PET/CT may be helpful in the management of patients with advanced basal cell carcinoma. Because it not only could assess the location and extent of skin lesions, but also early detect metastases [23, 24]. PET/CT be able to provide more information to clinicians to make therapeutic decisions. The patient in our report had the history of multiple basal cell carcinomas and left invasive breast cancer. If radiologists were aware of the link between various diseases, they would make the correct diagnosis of NBCCS and provide reasonable advice to clinicians. From a prognostic standpoint, early diagnosis and appropriate treatment are critical. If diagnosed, lifelong care with interdisciplinary medical collaboration is necessary [25].

In the case of presenting skin lesions, identification of cutaneous metastases originating from breast cancer and primary basal cell carcinomas was important for the patient’s therapeutic choices. Metastasis from internal malignant tumors to the skin was rare, with an incidence of 0.7–10%. Of all malignant tumors, breast cancer had the highest incidence of cutaneous metastases [26, 27]. Lookingbill et al. [28] reported that 237of 992 breast cancer patients (23.8%) had cutaneous metastases, which could occur via lymphatic or bloodstream transmission and were most common in the head, neck and trunk. We should be vigilant for non-healing ulcers, persistently hardening erythema and newly developed skin nodules [28, 29]. Previous studies suggested that these cutaneous metastatic lesions exhibit varying degrees of increased ^18^F-FDG metabolism [26, 27, 30]. In our case, the level of ^18^F-FDG metabolism in the treated breast cancer was lower than skin lesions, and no definite metastatic lesion was found at other site. Clinically, the levels of tumor markers (CEA, CA153 and CA125) were within the normal range. Therefore, we suspected that the skin lesions were the other primary tumors. Of course, pathological examination is necessary to identify primary and metastatic lesions. In addition, we also need to identify skin changes associated with breast cancer treatment. Skin toxicity changes associated with immunotherapy for renal cancer have been reported in the past, which closely correlate with the treatment history. The associated skin and subcutaneous lesions would subside after discontinuation of the drug [31].

In conclusion, NBCCS is a relatively rare syndrome, which have the features of developmental abnormalities and tumor susceptibility. Compared to conventional imaging, ^18^F-FDG PET/CT has more advantages in diagnosing of NBCCS. It is important to recognize this syndrome on ^18^FDG PET/CT because of different diagnoses and therapeutic consequences.

## Data Availability

The datasets generated during the current study were available from the corresponding author on reasonable request.

## Abbreviations

NBCCS: Nevoid basal cell carcinoma syndrome
GGS: Gorlin-Goltz syndrome
BCNS: Basal cell nevus syndrome
PET/CT: Positron emission tomography/computed tomography
CT: Computed tomography
MRI: Magnetic resonance imaging
SUVmax: Maximum standardized uptake value
CEA: Carcinogenic embryonic antigen
CA153: Cancer antigen153
CA125: Cancer antigen 125
